# Corrigendum

**DOI:** 10.1093/jxb/eru303

**Published:** 2014-08-21

**In *Posidonia oceanica* cadmium induces changes in DNA methylation and chromatin patterning**

**Maria Greco, Adriana Chiappetta, Leonardo Bruno and Maria Beatrice Bitont**

*Journal of Experimental Botany*, Vol. 63, No. 2, pp. 695–709, 2012, doi:10.1093/jxb/err313

In the above paper Table 1 was incorrectly formatted. The correct table is republished below:

**Table 1.** Band-profiles classes defined on the basis of methylation sensitivity and restriction pattern of isoschizomers *Hpa*II and *Msp*I HpaII and MspI recognize the same tetranucleotide sequence (5′-CCGG-3′), but display differential sensitivity to DNA methylation. Underlined cytosine is methylated.

	Methylation status	MSAP band profile		Sensitivities to site-specific methylation	
		*Hpa*II	*Msp*I	*Hpa*II	*Msp*I
Class I	CCGG CCGG	Present	Present	Active	Active
	GGCC GGCC				
Class II	CCGG	Present	Absent	Active	Inactive
	GGCC				
Class III	CCGG	Absent	Present	Inactive	Active
	GGCC				
Class IV	CCGG	Absent	Absent	Inactive	Inactive
	GGCC				

